# Divergence between bread wheat and *Triticum militinae* in the powdery mildew resistance *QPm.tut*-*4A* locus and its implications for cloning of the resistance gene

**DOI:** 10.1007/s00122-018-3259-3

**Published:** 2018-12-07

**Authors:** Eva Janáková, Irena Jakobson, Hilma Peusha, Michael Abrouk, Monika Škopová, Hana Šimková, Jan Šafář, Jan Vrána, Jaroslav Doležel, Kadri Järve, Miroslav Valárik

**Affiliations:** 1 Institute of Experimental Botany of the Czech Academy of Sciences, Centre of the Region Haná for Biotechnological and Agricultural Research, Šlechtitelů 31, 78371 Olomouc, Czech Republic; 2 Department of Chemistry and Biotechnology, Tallinn University of Technology, Akadeemia tee 15, 19086 Tallinn, Estonia; 3 Present Address: Limagrain Central Europe Cereals, s.r.o., Hrubčice 111, 79821 Bedihošť, Czech Republic; 4 Present Address: Biological and Environmental Science and Engineering Division, King Abdullah University of Science and Technology, Thuwal, 23955-6900 Kingdom of Saudi Arabia

## Abstract

A segment of *Triticum militinae* chromosome 7G harbors a gene(s) conferring powdery mildew resistance which is effective at both the seedling and the adult plant stages when transferred into bread wheat (*T. aestivum*). The introgressed segment replaces a piece of wheat chromosome arm 4AL. An analysis of segregating materials generated to positionally clone the gene highlighted that in a plant heterozygous for the introgression segment, only limited recombination occurs between the introgressed region and bread wheat 4A. Nevertheless, 75 genetic markers were successfully placed within the region, thereby confining the gene to a 0.012 cM window along the 4AL arm. In a background lacking the *Ph1* locus, the localized rate of recombination was raised 33-fold, enabling the reduction in the length of the region containing the resistance gene to a 480 kbp stretch harboring 12 predicted genes. The substituted segment in the reference sequence of bread wheat cv. Chinese Spring is longer (640 kbp) and harbors 16 genes. A comparison of the segments’ sequences revealed a high degree of divergence with respect to both their gene content and nucleotide sequence. Of the 12 *T. militinae* genes, only four have a homolog in cv. Chinese Spring. Possible candidate genes for the resistance have been identified based on function predicted from their sequence.

**Electronic supplementary material:**

The online version of this article (10.1007/s00122-018-3259-3) contains supplementary material, which is available to authorized users.

## Introduction

Disease imposes an important constraint on crop productivity and is most effectively and sustainably managed by breeding cultivars harboring genetic resistance. The ability of many pathogens to overcome host resistance means that the discovery of additional sources of resistance is a must for any crop improvement program. Since the gene pool of bread wheat (*Triticum aestivum*) has been so extensively narrowed by more than a century of intensive breeding (Feuillet et al. 2008), the search for novel resistance genes needs to be extended to older materials such as landraces and even to related cultivated and wild species (Mondal et al. 2016; Zamir 2001). Over many years, wheat cytogeneticists and breeders have succeeded in developing a diverse collection of germplasm harboring introgression segments of variable length, many of which have targeted the introduction of genes conditioning resistance to leaf pathogens (King et al. 2017; Valkoun 2001). Advances in genomic technologies have enabled a much greater precision than has been possible hitherto in the characterization of these materials (Abrouk et al. 2017; Tiwari et al. 2014; Winfield et al. 2016). A number of introgressed genes conferring resistance to a fungal pathogen have been successfully isolated in hexaploid wheat: these currently comprise two conferring resistance to *Puccinia triticina* (Huang et al. 2003; Thind et al. 2017), five to *P. graminis* (Mago et al. 2015; Periyannan et al. 2013; Saintenac et al. 2013; Steuernagel et al. 2016) and one to *Blumeria graminis* (Hurni et al. 2013). *B. graminis* (commonly referred to as powdery mildew) epiphytotics can induce significant yield losses (Conner et al. 2003; Leath and Bowen 1989). A number of major resistance genes (*Pm* genes) have been identified (www.wheat.pw.usda.gov/cgi-bin/GG3/browse.cgi?class=gene); as most of these confer race-specific resistance, they are prone to being overcome by the rapidly evolving pathogen. Often durable form of resistance, referred to as adult plant resistance (APR), is typically conditioned by multiple genes, each conferring a small, but cumulative effect. As a result, uncovering its genetic basis normally requires quantitative trait locus (QTL) analysis. A meta-analysis (Lillemo and Lu 2015) has revealed that such genes are dispersed over 24 QTL-harboring regions, located on 18 of the 21 wheat chromosomes.

The tetraploid bread wheat relative *T. militinae* (genome formula A^t^G) is generally considered to be a spontaneous mutant of *T. timopheevii* (Dorofeyev et al. 1976), although it has also been suggested to have arisen from an introgressive hybridization between *T. timopheevii* and the BA^u^ tetraploid *T. carthlicum* (Järve et al. 2002). Derivatives of a wide cross between the bread wheat cultivar (cv.) Tähti (genome formula BA^u^D) and *T. militinae* include a selection (line 8.1) which harbors *T. militinae* segments incorporated within chromosomes 1A, 1B, 2A, 4A, 5A, 5B and 7A (Jakobson et al. 2006). Among other traits introduced from *T. militinae,* line 8.1 exhibits a marked improvement in the level of powdery mildew resistance expressed at both the seedling and adult plant stages. According to a QTL analysis, the genetic basis of this resistance is dominated (respectively, 33% and 54% of the variance shown by seedlings and adult plants) by a genes within a segment located near the distal end of chromosome arm 4AL, referred to as *QPm.tut*-*4A* (Jakobson et al. 2006). When present in a cv. Tähti background, the resistance decreases the number of secondary haustoria formed by the pathogen and enhances host cell apoptosis (Islamov et al. 2015). The gene (or genes) present within *QPm.tut*-*4A*, which acts in a race non-specific manner (Jakobson et al. 2012), is located in *T. militinae* itself on chromosome 7G (Abrouk et al. 2017). In a hybrid between wild-type bread wheat and the introgression line, little recombination occurs between the *T. militinae* segment and the segment of chromosome 4AL which has been replaced in line 8.1. However, it has been possible to define the genetic length and position of the segment to a 2.5 cM interval (Jakobson et al. 2012). Here, the focus was to isolate the gene(s) which determine the *QPm.tut*-*4A* resistance. By deploying a range of genetic and genomic strategies, it has proved possible to identify a small number of candidate genes and to characterize and contrast the sequences which originated from chromosome 7G with those which they replaced on 4A.

## Materials and methods

### Plant materials and mapping populations

The Jakobson et al. (2012) mapping population comprised 98 F_2_ progeny bred from the cross cv. Chinese Spring (CS) × line 8.1; this was extended for the purpose of increasing the level of resolution by self-pollinating F_2_ individuals which were heterozygous for *QPm.tut*-*4A* through to the F_5_. In addition, a second mapping population (hereafter referred to as the “*ph1* population”) was created by crossing the CS *ph1b* mutant (Sears 1977) with T312.30.38.16, a derivative of introgressive line 8.1 in which the only *T. militinae* segment present was the one harboring *QPm.tut*-*4A.* In order to derive a parental plant which was both homozygous for the *ph1b* allele and heterozygous for the segment harboring *QPm.tut*-*4A*, the resulting F_1_ hybrid was back-crossed to the *ph1b* mutant. Marker-assisted selected BC_1_F_1_ individuals were then allowed to self-pollinate. Selection for the *ph1b* allele was performed using a multiplex PCR assay involving the markers AWJL3, PSR128, PSR2120 and PSR574 (Roberts et al. 1999), while the *QPm.tut*-*4A* harboring segment was marked by *owm76* and *owm96*. The subsequent generations (BC_1_F_3_ and further) were not employed for this study due to their low viability and fertility presumably caused by extensive chromosomal rearrangements associated with the action of *ph1*. Doubled haploid line DH397 containing the *T. militinae* resistance locus only on 4AL was derived from cross Tähti × 8.1 (Jakobson et al. 2012). A bacterial artificial chromosome (BAC) library was constructed from the DNA of 4AL telosomic chromosome flow sorted from 4AL ditelosomic line carrying the 4A *T. militinae* introgression (Jakobson et al. 2012). Grain of the CS aneuploid stocks nullisomic 4A-tetrasomic 4B and nullisomic 4A-tetrasomic 4D, required to validate the chromosome specificity of newly developed markers, was provided by the National BioResource Centre (Kyoto, Japan). A doubled haploid line (DH81) carrying the same *T. militinae* translocations which determined the powdery mildew resistance as line 8.1 (Jakobson et al. 2012) served as a further control. Finally, the bread wheat cv. Kanzler was used a susceptible host in experiments involving powdery mildew inoculations.

### Phenotyping for powdery mildew resistance

Seedling resistance to powdery mildew was scored using an assay based on detached first seedling leaves of 10-day-old plants. Each leaf was cut into four segments, each of which was then laid in a Petri dish containing 0.6% agar supplemented with 0.35% *w*/*v* benzimidazol. The leaf segments were inoculated with four different isolates (2.1, 9.8, 13 and 14), as described by Jakobson et al. (2012). The Petri dishes were held at 17.5 °C, and the response was evaluated after 10 days using the 0–9 scale devised by Lutz et al. (1992). For each self-pollinated recombinant line selected from F_2_-derived F_3-5_ families, up to 30 progeny homozygous in the *QPm.tut*-*4A* region were phenotyped. The resistance status of each recombinant line was verified by comparing its progeny scores with scores of 12–16 progeny of homozygous nonrecombinant sister line selected from the same self-pollination.

### The development of markers used for high-density genetic mapping

The informativeness of a potential marker was first assessed via an *in silico* inspection of the chromosome-specific survey sequences of 4AL-7G (Abrouk et al. 2017) and the chromosome survey sequences (CSS) of 4AL^CS^ generated by IWGSC (2014). The choice of sequences was governed by the need to saturate the genetic map of the *QPm.tut*-*4A* region, so it was based on a virtual gene order of the chromosome 4A represented by GenomeZippers (Abrouk et al. 2017, Hernandez et al. 2012). 4AL CSS scaffolds obtained from the GenomeZipper delimited by the markers flanking the *QPm.tut*-*4A* segment were aligned with 4AL-7G sequence scaffolds using the BlastN algorithm (Altschul et al. 1990). Only low-copy sequences associated with a nucleotide identity of > 95% were considered for marker development, and those containing short indels were preferred. Where no indel was identifiable, markers were based on single-nucleotide polymorphisms using the cleaved amplified polymorphic sequence approach (Michaels and Amasino 1999). PCR primers were designed using Primer3 software (Untergasser et al. 2012). To ensure specificity for 4AL, given the presence of homoeologous sequence on 7AS and 7DS, primers were positioned to ensure the presence of a variant nucleotide close to 3′ end in the homoeologous sequences. Finally, the specificity of the putative amplicons was verified by a BlastN search against the whole wheat genome CSS (IWGSC 2014). A second marker discovery strategy profited from a BAC library-based, established CS 4AL-specific physical map (IWGSC 2018, URGI; urgi.versailles.inra.fr/). BAC clones making up the set of contigs which covered the *QPm.tut*-*4A* region were selected from the minimal tiling path and sequenced. Subsequently, sequence scaffolds positioned at the target location were employed for marker development based on the first strategy. A final strategy designed to extend the saturated portion of the *QPm.tut*-*4A* region beyond what was achievable using the first two strategies was based on the sequence of the *T. dicoccoides* 7AS region (Avni et al. 2017) and on the CS reference sequence WGA v0.4 (www.wheatgenome.org); this also was informative for identifying additional 4AL^CS^ scaffolds for targeted marker development. All primer pairs were tested on a template of CS, DH81, a CS/line 8.1 derivative heterozygous for *QPm.tut*-*4A*, nullisomic 4A-tetrasomic 4B and nullisomic 4A-tetrasomic 4D and DNA from flow-sorted chromosome arms 4AL^CS^ and 4AL-7G amplified according to Šimková et al. (2008). The methods used for PCR amplification and electrophoretic separation are given in the following section, and the primer sequences and associated information are summarized in Table S1. Where the physical position of a marker was uncertain, it was determined by screening 62 three-dimensional pools prepared from the minimal tiling path of the CS 4AL-specific physical map.

### Genetic mapping

DNA was extracted using Agencourt^®^ Genfind^®^ v2 magnetic beads (Beckman Coulter Life Sciences, Indianapolis, IN, USA) on a Beckman Coulter^®^ Biomek^®^ NX^P^ workstation, as described by Ivaničová et al. (2016). Markers *owm82* and *Xgwm160* (Röder et al. 1998) were used to select lines in which a recombination event had occurred in the region of the introgression segment in the mapping populations. The full set of markers was applied to genotype those arising from the CS × line 8.1 population, while a subset of 19 selected markers was applied to those arising from the *ph1* population. Each 15 µL PCR contained 0.01% (*w*/*v*) o-cresolsulphonephtalein, 1.5% (*w*/*v*) sucrose, 0.2 mM of each dNTP, 0.6 U Taq DNA polymerase, 1 µM of each primer, 10 mM Tris-HCl, 50 mM KCl, 1.5 mM MgCl_2_ and 0.1% (*v*/*v*) Triton X-100. The template comprised either 10–20 ng genomic DNA or 5 ng DNA amplified from 4AL^CS^ or 4AL-7G. The reaction conditions consisted of an initial denaturation step of 95 °C/5 min, followed by 40 cycles of 95 °C/30 s, an optimized annealing temperature (Table S1) for 30 s and 72 °C for 30 s per 500 bp amplicon length; the reactions were completed with an elongation step of 72 °C/5 min. Cleaved amplified polymorphic sequence assays were completed with a digestion using the appropriate restriction endonuclease. The amplicons were electrophoretically separated through 4% non-denaturing polyacrylamide gels and visualized by ethidium bromide staining.

### Construction of the physical map

Sequence scaffolds taken from IWGSC RefSeq v1.0 (IWGSC 2018) were used to span the genomic region of CS 4AL replaced by the *QPm.tut*-*4A* segment in line 8.1. To obtain the corresponding sequence from *T. militinae*, a chromosome walking approach was initiated. A chromosome-specific BAC library designated TaaPmt4ALhA (www.olomouc.ueb.cas.cz/dna-libraries/cereals) was constructed from flow-sorted 4AL-7G chromosome arms according to the Šimková et al. (2011) protocol. BAC library plate pools were generated by mixing 40 µL of the bacterial culture from each well of a given plate, centrifuging (10 min at 2700 g), suspending the precipitate in 0.5 mL TE and boiling for 30 min. The suspension was then re-centrifuged (60 min at 2700 g), and a 450-µL aliquot of the supernatant was diluted 100-fold. The subsequent PCRs used as template a 1.5-µL aliquot of the diluted plate pool DNA. The plate pools were screened with the markers *owm169* and *owm228,* together with other codominant markers located within the CS 4AL segment replaced by *QPm.tut*-*4A* (*owm156*, *owm136*, *owm221*, *owm209*, *owm227*, *owm236*, *owm139* and *owm235*). Row and column pools of positive plates were prepared and used in the same way as plate pools. Selected BAC clones were sequenced on a MiSeq instrument (Illumina Inc., San Diego, CA, USA) using a Nextera DNA Library Prep Kit (Illumina) according to the manufacturer’s protocol. Paired-end reads were assembled by Ray software (Boisvert et al. 2010). Insertion site-based polymorphism or site-specific presence/absence variation markers were developed from the ends of sequenced BAC clones and used for a further round of BAC library screening. The procedure was iterated until the physical map had been assembled.

### The identification and sequence analysis of candidate genes

The gene content along the *QPm.tut*-*4A* segment on 4AL-7G was annotated using the TriAnnot pipeline (Leroy et al. 2011, www6.inra.fr/decodage/TriAnnot). Predicted genes were subjected to a BlastP search against the set of non-redundant protein sequences (www.ncbi.nlm.nih.gov/BLAST), and conserved domains of putative proteins were annotated using the NCBI Conserved Domain Database (Marchler-Bauer et al. 2017) and the MOTIF search tool (www.genome.jp/tools/motif/) based on the Pfam database (Finn et al. 2016). Searches for homologs and the comparison of candidate gene sequences were based on the BlastN algorithm, applying a threshold of 90% identity and 60% coverage.

### Reverse transcription PCR

Segments of the first leaf of 10-day-old seedlings of the doubled haploid line DH397 and line T312.30.38.16 were collected 0-, 24- and 48-h post-inoculation with powdery mildew and snap frozen in liquid nitrogen. Leaf segments from three independent inoculations were bulked for the purpose of RNA extraction, and negative control samples were formulated from non-inoculated leaf segments. The leaf tissue was homogenized using ball mill MM 301 (Retsch, Haan, Germany) with three 3 mm tungsten beads at 30 Hz for 45 s, and total RNA was extracted using a miRNeasy Mini Kit (Qiagen Inc., Hilden, Germany) according to the manufacturer’s protocol. The synthesis of the first cDNA strand was achieved using a Transcriptor High Fidelity cDNA Synthesis Kit (Roche Life Sciences, Indianapolis, IN, USA) based on an anchored-oligo(dT)_18_ primer. Gene-specific primers were designed for all 12 candidate genes such that the amplicons derived from gDNA differed in length from those derived from cDNA (Table S2). A portion of the *Actin* gene (GenBank accession number AB181991.1) was used as the reference sequence. The procedures used for PCR and electrophoretic separation were as described above.

## Results

### High-resolution genetic mapping around QPm.tut-4A

**Fig. 1:**
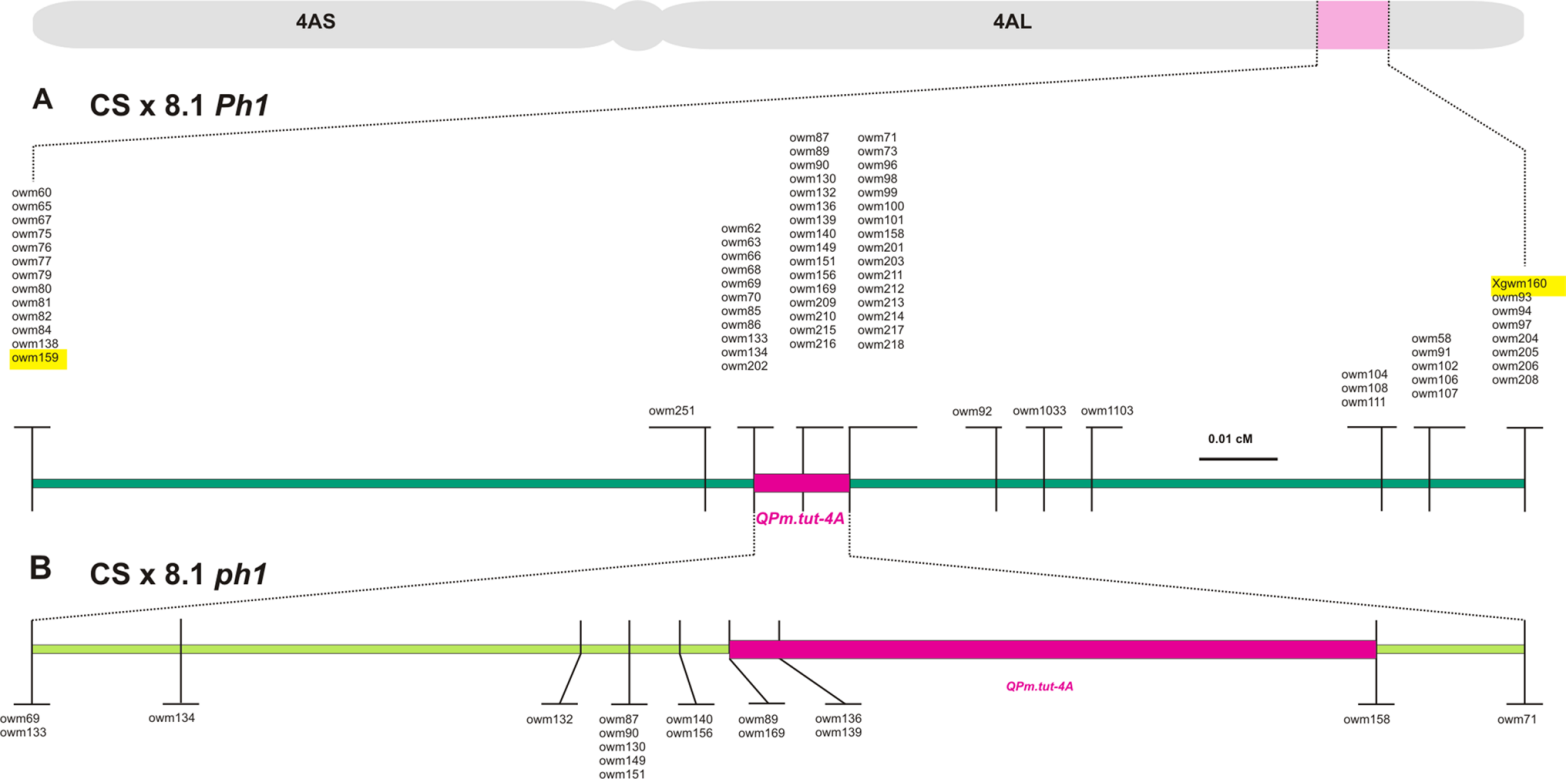
A high-density genetic map of the region harboring *QPm.tut*-*4A*. **a** Marker segregation displayed by 8519 progeny bred from the CS × 8.1 mapping population (including the F_2_ generation described at Jakobson et al. 2012) defines the genetic length of the *QPm.tut*-*4A* segment (marked in red) to 0.012 cM, lying within a 0.18 cM window within chromosome 4AL. The flanking markers used for identification of recombination events within the segment are highlighted in yellow. **b** The level of mapping resolution achieved was enhanced through the use of the *ph1* mapping population, which provided an additional 30 new recombination events, thereby splitting the *QPm.tut*-*4A* region into eight subregions

**Fig. 2:**
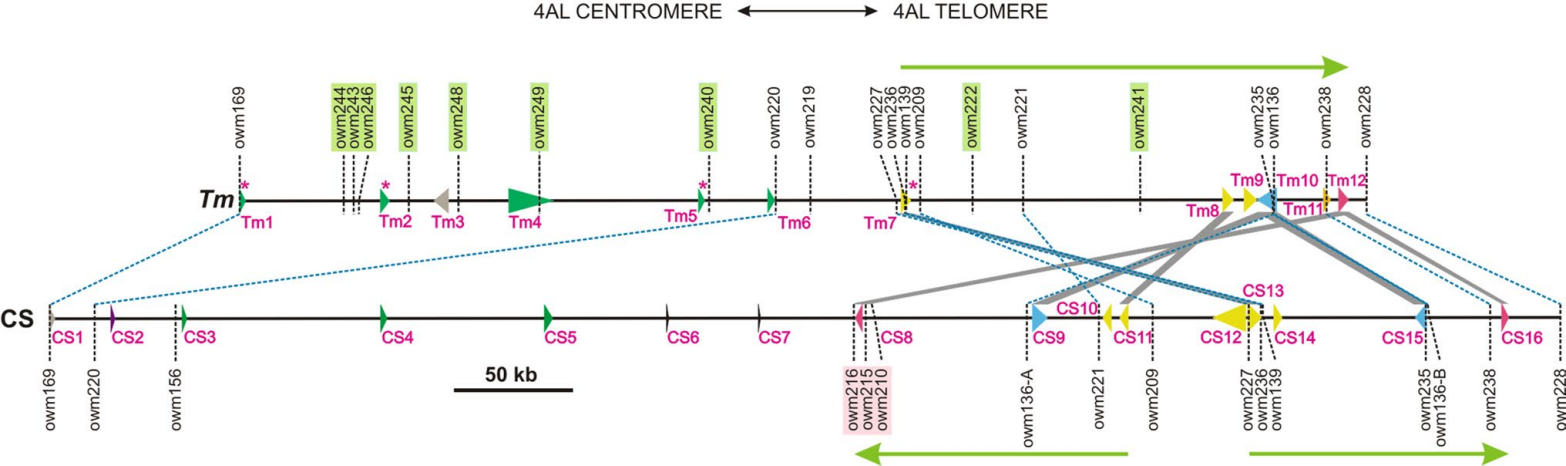
Physical maps showing the gene content of the 480.2 kbp *QPm.tut*-*4A* segment inherited from *T. militinae* and the 640.8 kbp segment of chromosome 4AL replaced by the introgression in line 8.1. The predicted genes (*Tm1*–*Tm12* and *CS1*–*CS16*) are depicted by arrowheads indicating their orientation. The size of the arrowheads is proportional to the length of the gene, and their color reports the presence of conserved domains: green: CC-(LRR)-NB-ARC; yellow: PGG; orange: ANK and PGG; blue: LRR/MAL/PK; red: patatin-like; black: PMEI-like; and gray: uncharacterized protein. *Tm* and *CS* genes linked by gray lines share sequence identity in their coding region of at least 90% at a coverage of ≥ 60% (a coverage < 80% applied only to the putative pseudogene *CS15*). *Tm* genes which produced a detectable transcript are indicated by a red asterisk. Shared markers are linked by a blue dashed line, those marking *T. militinae* but not CS genomic sequence, or *vice versa,* are highlighted in, respectively, light green and light red. The *T. militinae* region bounded by *owm227* and *owm228* was present in CS as two tandemly arranged, tail-to-tail orientated copies, as indicated by the green arrows

A set of 102 new genetic markers was developed to saturate the genetic map in the region of *QPm.tut*-*4A* (Figs. 1, 2, Table S1). Majority of these were developed using GenomeZipper and 4AL-specific BAC clones. Besides markers *owm16* and *owm39*, polymorphism was inferred using the 4AL-7G and 4AL^CS^ survey sequences (Abrouk et al. 2017, IWGSC 2014). Segregation patterns observed in the extended CS × 8.1 mapping population were used to establish marker order. The genotyping of 8425 individuals with respect to *owm82* and *Xgwm160* revealed 30 new recombination events. Testing these recombinant individuals for their reaction to the four powdery mildew isolates showed that the resistance phenotype was fully correlated with the presence of the *T. militinae* segment—the disease scores for the four isolates were, respectively, 0.5, 0.4, 0.1, 1.1 in the presence of the segment, and 3.3, 2.5, 2.6, 3.8 in its absence. The net effect of the mapping was reducing the genetic length of the segment harboring *QPm.tut*-*4A* to 0.012 cM (Fig. 1). The *ph1* population was derived from the self-pollination of 22 (out of 107 screened) BC_1_F_1_ ([line 8.1 × *ph1b*] × *ph1b*) selections which were simultaneously homozygous for the *ph1b* allele and heterozygous for the *QPm.tut*-*4A* segment. The genotyping of the resulting 1255 BC_1_F_2_ progeny revealed 155 recombination events between *owm82* and *Xgwm160*, equivalent to a 33-fold increase in recombination as compared to the rate observed in the presence of *Ph1.* The 0.012 cM *QPm.tut*-*4A* region was thereby split into eight subregions (Fig. 1b).

### The physical map of the QPm.tut-4A segment

Six additional markers for the *QPm.tut*-*4A* region were developed by inspection of the three CS reference sequence IWGSC WGA v0.4 scaffolds (134864, 108602 and 47761) (www.wheatgenome.org) selected on the basis of markers lying within the *owm169*–*owm158* interval (Table S1). The *owm228* marker was derived from same scaffold (134864) which contained the *QPm.tut*-*4A* flanking marker *owm169*. The application of these markers to the set of recombinant segregants defined the physical length of the CS sequence replaced by the *QPm.tut*-*4A* introgression to 640.8 kbp (Fig. 2). A 4AL-7G-specific BAC library was constructed in an effort to acquire sequences in the *QPm.tut*-*4A* region of line 8.1; the mean insert size harbored by the 43,008 clones generated provided a 6.8-fold coverage of the 4AL-7G chromosome arm (www.olomouc.ueb.cas.cz/dnalib/taapmt4alha). Pools of the set of 112 plates were assembled to perform a PCR screen based on the flanking markers *owm169* and *owm228*, along with eight codominant markers (*owm156*, *owm136*, *owm221*, *owm209*, *owm227*, *owm236*, *owm139* and *owm235*) mapping within the region (Fig. 2). One to seven positive BAC clones per marker were sequenced so that their end sequences could be used for the next round of chromosome walking-based marker development: 17 new markers were designed in this way (Table S1). In total, 26 BAC clones were sequenced to provide a complete sequence of the *QPm.tut*-*4A* region in line 8.1: The physical length of the segment bounded by *owm169* and *owm228* was 480.2 kbp.

### Annotation and comparative analysis of the QPm.tut-4A segment

The *T. militinae* region was 25% shorter than the CS one (480.2 vs. 640.8 kbp). Gene annotation suggested that the *QPm.tut*-*4A* segment harbored 12 high-confidence (HC) protein-encoding genes (denominated *Tm1*–*Tm12*, Fig. 2, Table 1), while the 4AL region of CS harbored 16 HC genes (*CS1*–*CS16*, Table 2). The intergenic regions displayed no sequence similarity. A comparison of the *T. militinae* and *CS* genes based on a threshold of 90% identity and 60% coverage implied that only four of the *T. militinae* genes (*Tm7*, *Tm8*, *Tm10* and *Tm12*) shared an appreciable level of homology with members of the *CS* set (*CS8*, *CS9*, *CS11*, *CS13*, *CS15* and *CS16*) (Table 1, Fig. 2). The sequence of the proximal ends of the CS and line 8.1 regions (beyond *owm169*) were quite distinct with respect to both their sequence and gene content: The segment from *owm169* to *owm220* was 228 kbp in line 8.1, but only 19 kbp in CS, and they contained, respectively, six (*Tm1*–*Tm6*) and one (*CS1*) genes. The central part of the segment (*owm220*–*owm227* in line 8.1 and *owm220*–*owm216* in CS) was longer in CS (322 vs. 51 kbp) and contained no predicted gene in line 8.1 and *CS2*–*CS7* in CS. The distal region (*owm227*–*owm228* in line 8.1 and *owm216*–*owm228* in CS) was, respectively, of length 200 kbp (genes *Tm7*–*Tm12*) and 299 kbp (*CS8*–*CS16*) (Fig. 2). The proximal segment harboring *Tm1*–*Tm6* was not represented on CS chromosome 4A. However, four of these genes (*Tm1*, *Tm2, Tm5* and *Tm6*) each have a strong homoeolog (Table 3) lying in reverse orientation within a ~ 410 kbp region of chromosome arm 7AS situated about 17.4 Mbp from the telomere. The position of the *QPm.tut*-*4A* region is 41.05 Mbp distant from the 4AL telomere. The distal part of the region (*owm227*–*owm228*) has been duplicated in CS, and the gene content has been differentiated. The two copies are arranged tandemly in a tail-to-tail orientation, resulting in the non-syntenic location of the *owm227* marker (Fig. 2).

**Table 1:** Predicted candidate genes present in the *QPm.tut*-*4A* segment introgressed from *T. militinae* into line 8.1

No.	Putative conserved protein domains	No. of exons	Length of protein sequence (aa)	CS homologous genes in the QPm.tut-4A region^a^	Identity (%)	Coverage (%)	RNA expression^b^
*Tm1*	CC; NB-ARC	2	225	–	–	–	+
*Tm2*	CC; LRR; NB-ARC	2	753	–	–	–	+
*Tm3*	–	3	253	–	–	–	–
*Tm4*	CC; LRR; NB-ARC	7	894	–	–	–	–
*Tm5*	CC; LRR; NB-ARC	2	557	–	–	–	+
*Tm6*	CC; LRR; NB-ARC	2	1017	–	–	–	–
*Tm7*	5× PGG	2	963	TraesCS4A01G450100.2 (CS13)	97	99	+
*Tm8*	5× PGG	3	764	TraesCS4A01G449900 (CS11)	90	98	–
*Tm9*	4× PGG	2	849	–	–	–	–
*Tm10*	LRR; Malectin; Pkinase	23	913	TraesCS4A01G449700 (CS9) TraesCS4A01G450300 (CS15)	97 98	84 60	–
*Tm11*	Ankyrin repeats; PGG	4	636	–	–	–	–
*Tm12*	Patatin-like phospholipase	5	424	TraesCS4A01G449600 (CS8) TraesCS4A01G450400 (CS16)	98 97	100 100	–

*CC* coiled coil, *NB-ARC* nucleotide-binding adaptor shared by APAF-1, certain *R* gene products and CED4, *LRR* leucine-rich repeat, *PGG* proline-glycine-glycine (domain named for this highly conserved sequence motif found at its start), *Pkinase* protein kinase

^a^Acronym for each gene used in the body of this manuscript and Fig. 2 is provided in the parentheses

^b^Detected in first leaves of DH397 and T312.30.38.16 seedlings

**Table 2:** Gene content of the segment of CS chromosome arm 4AL replaced by the *QPm.tut*-*4A* segment introgressed from *T. militinae* into line 8.1

No.	Official designation^a^	Putative conserved protein domains	No. of exons	Length of protein sequence (aa)
*CS1*	TraesCS4A01G448900	CC	3	313
*CS2*	TraesCS4A01G449000	Transferase	1	472
*CS3*	TraesCS4A01G449100	CC; NB-ARC	2	353
*CS4*	TraesCS4A01G449200	NB-ARC; LRR	2	838
*CS5*	TraesCS4A01G449300	CC; LRR; NB-ARC	3	1008
*CS6*	TraesCS4A01G449400	Plant invertase/pectin methylesterase inhibitor	1	203
*CS7*	TraesCS4A01G449500	Plant invertase/pectin methylesterase inhibitor	1	206
*CS8*	TraesCS4A01G449600	Patatin-like phospholipase	5	424
*CS9*	TraesCS4A01G449700	LRR; Malectin; Pkinase	21	867
*CS10*	TraesCS4A01G449800	5× PGG	2	993
*CS11*	TraesCS4A01G449900	5× PGG	2	1020
*CS12*	TraesCS4A01G450000	4× PGG	3	761
*CS13*	TraesCS4A01G450100.2	5× PGG	3	984
*CS14*	TraesCS4A01G450200	5× PGG	2	1024
*CS15*	TraesCS4A01G450300	LRR; Malectin	15	549
*CS16*	TraesCS4A01G450400	Patatin-like phospholipase	5	424

^a^The International Wheat Genome Sequencing Consortium RefSeq v1.0 annotation (IWGSC 2018)

**Table 3:** Homoeologs on CS chromosome arm 7AS of the NLR family genes present in the *QPm.tut*-*4A* segment introgressed from *T. militinae* into line 8.1

No.	CS homoeolog on 7AS	Identity (%)	Coverage (%)	Putative conserved protein domains of homoeologs	Length of protein sequence (aa)	Length difference relative to *T. militinae* homoeolog (aa)
*Tm1*	TraesCS7A01G038800	95	100	CC; NB-ARC	452	+ 227
*Tm2*	TraesCS7A01G039300	97	100	CC; LRR; NB-ARC	972	+ 219
*Tm4*	–	–	–	–	–	–
*Tm5*	TraesCS7A01G03910	98	100	CC; LRR; NB-ARC	1074	+ 517
*Tm6*	TraesCS7A01G039400	93	79	CC; LRR; NB-ARC	875	− 142

### Functional characterization of the candidate genes for powdery mildew resistance

The five genes *Tm1*, *Tm2* and *Tm4*–*Tm6* each encoded a coiled-coil (CC) domain and a nucleotide-binding (NB) domain, thereby being members of the NB-ARC family (van der Biezen and Jones 1998). *Tm2*, *Tm4* and *Tm6* also encode a leucine-rich repeat (LRR) domain, indicating them as members of the disease resistance-associated NLR gene family (Ye and Ting 2008). The shorter length of both *Tm1* and *Tm5* (Table 1) implies that both are incomplete genes. *Tm10* is predicted to encode a protein harboring an LRR/malectin/protein kinase (LRR/MAL/PK) domain, also shared by a number of plant disease resistance gene products (Sekhwal et al. 2015); this gene lies in the distal part of the introgression segment and shares homology with both *CS9* and the truncated *CS15* (Fig. 2). The sequences of the other six *Tm* genes have little or no connection with disease resistance: *Tm7*–*Tm9* each encode multiple PGG domains, *Tm11* features ankyrin repeats, while *Tm12* encodes a protein belonging to the patatin-like phospholipase family (Table 1). Four of the 12 *Tm* genes (*Tm1*, *Tm2*, *Tm5* and *Tm7*) produced a detectable level of mRNA, and three of them (*Tm1*, *Tm2* and *Tm5*) encode NLR proteins: *Tm2* appears to be the only one of these which is intact.

## Discussion

The introduction into the bread wheat gene pool of genes harbored by species belonging to its secondary and tertiary gene pools represents an attractive strategy for broadening the genetic diversity of a crop which has been intensively bred for over a century. However, the success of the strategy has been not infrequently limited by the simultaneous introgression of linked genes which are deleterious to either productivity and/or product quality. This phenomenon of linkage drag results from the suppression of recombination around an introgressed segment, a phenomenon which also hampers positional cloning, since it magnifies the ratio between physical (in bp) and genetic (in cM) distance and prevents efficient high-density mapping. The *T. militinae* segment harboring *QPm.tut*-*4A* suffers from exactly this problem. Recent significant progress in the next-generation sequencing technologies allowed development of two new gene cloning approaches called MutRenSeq (Steuernagel et al. 2016) and MutChromSeq (Sánchez-Martín et al. 2016) which can bypass the high-density map construction in gene cloning process. The MutRenSeq approach employing exome capture and sequencing focusses on identification of simultaneous deleterious mutations in single NB-LRR-like gene within mutants with lost resistance. The MutChromSeq uses similar approach consisting in identification of knockout mutations in single gene within mutant lines, but it requires flow sorting of respective chromosomes from all mutant lines and their sequencing. This means that only single dominant major-effect genes can be identified by these approaches. However, the *QPm.tut*-*4A* locus is associated with race non-specificity and incomplete resistance which suggests the resistance may be encoded by gene different from major-effect *R* genes (predominantly NB-LRR-like genes) or by more than one gene (Jakobson et al. 2006, 2012). The MutRenSeq and MutChromSeq approaches are therefore not feasible for the *QPm.tut*-*4A* gene/genes cloning. Fortunately, recent advances in DNA technology and the development of sophisticated genomic resources have substantially eased the processes of constructing a high-density map and of acquiring relevant sequence from both the donor and the recipient genomes.

### High-density mapping

According to the Wheat-Composite2004-4A map (wheat.pw.usda.gov/GG3), the markers flanking *QPm.tut*-*4A* (*Xwmc232* and *Xgwm160*) are separated by ~ 9 cM, which led Jakobson et al. (2012) to suggest that a plant heterozygous for the introgression experiences suppression of recombination in the region, as has been documented for a number of other bread wheat introgression segments (Bariana et al. 2001; Järve et al. 2000; Jia et al. 1996; Lukaszewski 2015). Several genomic resources developed in CS were exploited here to saturate the region with markers in order to fine map the resistance locus, and these greatly enhanced the efficiency with which such markers could be elaborated, especially compared to conventional approaches to marker discovery such as microsatellite screening (Röder et al. 1998) or searching for sequence polymorphism in expressed sequence (e.g. Valárik et al. 2006). The GenomeZipper and chromosome-specific survey sequences (Abrouk et al. 2017, Hernandez et al. 2012, IWGSC 2014) were particularly effective in this context, but use was also made of sequence scaffolds developed in the tetraploid wheat *T. dicoccoides* (Avni et al. 2017) and the BAC contigs defining the chromosome 4AL physical map (IWGSC 2018). Initially, these resources allowed the genetic length of the target to be narrowed to just 0.012 cM (Fig. 1). In a fully homologous situation, such as occurs on wheat chromosome 3B, the mean ratio between cM and Mbp in distal regions varies from 0.60 to 0.96 (Choulet et al. 2014). If the recombination between the introgressed segment and the unaltered bread wheat region was unimpeded, the physical length of the segment would have been estimated to be no longer than 20 kbp. The fact that the segment’s length was measured in hundreds of kbp demonstrated that there was a substantial localized suppression of recombination.

### Structural divergence and recombination in the QPm.tut-4A segment

The pairing of homoeologs in hexaploid wheat is strongly restricted by the action of the *Ph1* locus (Riley and Chapman 1958). The intention of creating the *ph1* population was to induce a higher rate of recombination between the introgression containing *QPm.tut*-*4A* and its presumed homoeologous segment of CS 4AL, since the rate of recombination achieved in the presence of *Ph1* was < 0.4% (31 out of 8519) within the *owm82*-*Xgwm160* region (Fig. 1): Although this represented a very small genetic distance (0.18 cM), the suppression of pairing/recombination meant that the physical distance involved was potentially rather large. The effect was enhancing the rate of recombination by 33-fold. A comparable elevation in the recombination rate induced by removal of the *Ph1* locus has been reported in a variety of interspecific hybrids (Lukaszewski 1995, Luo et al. 2000). The availability of the genomic sequence of CS (IWGSC 2018) made it possible to identify that the length of the 4AL segment (0.012 cM) replaced by the *owm169*-*owm228 QPm.tut*-*4A* locus in line 8.1 was 640 kbp (Figs. 1, 2) and that it harbors 16 predicted genes (Table 2). The inferred relationship between genetic and physical distance in the segment was therefore only 0.019 cM per Mbp, which is much lower than the ratio (0.60–0.96 cM per Mbp) obtained in the distal region of chromosome 3B and is even below the ratio associated with the 3B centromeric region (0.05 cM per Mbp) in which recombination is known to be repressed (Choulet et al. 2014). This major suppression of recombination reflects the lack of homology between the native wheat and the introgressed *QPm.tut*-*4A* locus. The length of the introgressed *QPm.tut*-*4A* locus (*owm169*–*owm228*) was 480 kbp (Fig. 2), and it harbored 12 predicted genes (Table 1), eight of which lacked a homolog in the CS segment (Table 1, Fig. 2). The CS homologs of the other four genes (*Tm7*, *Tm8*, *Tm10* and *Tm12*) lay in a duplicated segment, and one of the duplicated copies was present in inverted orientation. The region was further disrupted by a number of indels, and all these changes suggest a high level of evolutionary dynamics of the wheat genome. While loss of synteny and sequence divergence are commonplace between homoeologous genomes (Saintenac et al. 2013; Wicker et al. 2003), they can also feature in comparisons made between homologous genomes of different hexaploid wheat cultivars (Mago et al. 2014; Tsõmbalova et al. 2016).

The introgressed *QPm.tut*-*4A* locus included three full-length and two truncated NLR family genes (Table 1). This class of genes is frequently arranged in clusters, which is thought to reflect the outcome of duplication events followed by sequence divergence (Michelmore and Meyers 1998). The coding sequences of the *T. militinae* NLR-like genes shared only a moderate to a high (73–89%) level of identity at the nucleotide level. Their putative homoeologs present on chromosome arm 7AS of CS displayed a similar level of sequence relatedness (71–85%). This chromosomal location confirms the conclusion of Abrouk et al. (2017) that in *T. militinae* itself, the *QPm.tut*-*4A* segment is present on chromosome 7G. The G genome donor is thought to be a member of the *Sitopsis* section of the genus *Aegilops,* as is also the donor of the bread wheat B genome (Gornicki et al. 2014). The bread wheat chromosome 4A itself is a restructured chromosome composed of a mosaic of segments derived from 4AL, 5AL and 7BS (Devos et al. 1995, Hernandez et al. 2012), and the *QPm.tut*-*4A* introgression appears to lie within a part of this region which originated from 7BS, consistent with its transfer following meiotic pairing.

### The potential function of the QPm.tut-4A candidates

The resistance to powdery mildew associated with the presence of the *QPm.tut*-*4A* segment was race non-specific and in a cv. Tähti background, accounted for 40% of the variation in resistance (Jakobson et al. 2012). To date, only few wheat genes associated with APR have been isolated, so unlike the case for race-specific seedling resistance genes, many of which belong to the NLR family, it is not clear what functionality the product of such a gene might have. The *Lr34/Yr18/Pm38* gene, which provides protection against three distinct foliar pathogens, has been shown to encode an ABC transporter (Krattinger et al. 2009), while *Lr22a*, which confers broad-spectrum APR to leaf rust, encodes an NLR-like protein (Thind et al. 2017), as does the rice NLR family *Pb1* gene against panicle blast (Hayashi et al. 2010). Assuming that one of the three NLR family genes *Tm2*, *Tm4* and *Tm6* represents the most likely candidate for the *QPm.tut*-*4A* resistance, the possible basis of its resistance being race non-specific needs to be explored. In one scenario, it may be that either two or even all three of the *Tm* genes, which are each individually race-specific, act together to confer apparent race non-specificity. Alternatively, it is possible that one of the three genes is a “defeated” major resistance gene which has retained some residual broad-spectrum effect, as has been demonstrated for some other defeated major resistance genes (Li et al. 1999). The latter hypothesis is probably the more plausible, given that the only gene for which a mRNA was detected was *Tm2,* although surprisingly it was possible to detect transcript of the two NLR-like pseudogenes *Tm1* and *Tm5*. However, the lack of an LRR domain in the *Tm2*-encoded protein implies that it would be difficult for its product to recognize the pathogen’s avirulence signal. An additional candidate is represented by *Tm10* which encodes a protein containing an LRR, a MAL and a PK domain. A barley protein of this domain composition (HvLEMK1) is known to mediate non-host resistance to powdery mildew, while its wheat ortholog acts to enhance the level of wheat host resistance to powdery mildew (Rajaraman et al. 2016). A comparison of the TaLEMK1 and the *Tm10* protein sequences showed that they share only a 34% level of identity; this rather low level of homology, combined with the observations that the wheat *LEMK1* homoeologs map to the group 5 chromosomes and that no *Tm10* transcript was detected, rules out the possibility that the *QPm.tut*-*4A* resistance is conferred by *Tm10*. None of the other *Tm* genes encode a product which has been directly associated with disease resistance to date. However, *Tm12*—which encodes a protein belonging to the patatin-like phospholipase family—remains a candidate since the patatin-like protein AtPLP2 has been shown to represent a component of the cell machinery delivering apoptosis, and therefore makes a contribution toward resistance against an obligate biotroph (La Camera et al. 2009).

#### Author contribution statement

KJ, JD and MV designed the study; MV, EJ, MŠ, HŠ, JŠ and IJ were responsible for marker development, genotyping, BAC library construction, chromosome sorting, data analysis and the construction of the mapping populations. HP performed the phenotypic evaluation and IJ the statistical analysis. The bioinformatics analyses were conducted by EJ and MA, who also contributed to data interpretation. Other experiments were conducted by EJ. The manuscript was drafted by EJ and MV, and all the authors contributed to its editing and proofreading.

## Electronic supplementary material

Below is the link to the electronic supplementary material.

Supplementary material 1 (XLSX 33 kb)

Supplementary material 2 (XLSX 10 kb)
